# Lung Elastance and Microvascularization as Quantitative Non-Invasive Biomarkers for the Aetiological Diagnosis of Lung Consolidations in Children (ELASMIC Study)

**DOI:** 10.3390/diagnostics15070910

**Published:** 2025-04-02

**Authors:** Sergi Huerta-Calpe, Carmina Guitart, Josep Lluis Carrasco, Bárbara Salas, Francisco José Cambra, Iolanda Jordan, Mònica Balaguer

**Affiliations:** 1 Paediatric Intensive Care Unit, Hospital Sant Joan de Déu, Universitat de Barcelona (UB), Passeig Sant Joan de Déu, 2, 08950 Barcelona, Spain; sergi.huerta@sjd.es (S.H.-C.); carmina.guitart@sjd.es (C.G.); franciscojose.cambra@sjd.es (F.J.C.); monica.balaguer@sjd.es (M.B.); 2 Immunological and Respiratory Disorders in the Paediatric Critical Patient Research Group, Institut de Recerca Sant Joan de Déu (IRSJD), c. de Sta. Rosa, 39, 08950 Barcelona, Spain; 3 Escola de Doctorat Universitat de Barcelona (EDUB), c. Casanova, 143, 08036 Barcelona, Spain; 4 Biostatistics Department of Basic Clinical Practice, Universitat de Barcelona (UB), c. Casanova, 143, 08036 Barcelona, Spain; jlcarrasco@ub.edu; 5 Radiology and Diagnostic Imaging Unit, Hospital Sant Joan de Déu, Universitat de Barcelona (UB), Passeig Sant Joan de Déu, 2, 08950 Barcelona, Spain; barbara.salas@sjd.es; 6 Faculty of Medicine, Universitat de Barcelona (UB), c. Casanova, 143, 08036 Barcelona, Spain; 7 Faculty of Medicine, University of Vic-Central University of Catalonia (UVic-UCC), 08500 Vic, Spain

**Keywords:** lung consolidation, bacterial pneumonia, atelectasis, shear wave elastography, superb microvascular imaging, pulmonary elastance, pulmonary microvascularization ratio, paediatrics

## Abstract

**Background:** Acute lower respiratory tract conditions are highly prevalent in paediatrics. Many of these conditions present as consolidations on imaging studies. One of the most common causes is bacterial pneumonia (BP), which requires an accurate diagnosis to implement the best treatment plan. Despite the fact that major guidelines constrain the use of invasive tests, chest X-ray (CXR) or blood tests are still routinely used for the diagnosis. In this regard, the introduction of lung ultrasound (LUS) signified an advancement in reducing the invasiveness of diagnosis. However, there are still situations where distinguishing between different aetiologies remains challenging, especially in the case of atelectasis. **Methods:** This is a prospective cohort study to assess the diagnostic accuracy of new non-invasive, quantifiable, and reproducible imaging biomarkers (lung elastance and microvascularization ratio) for differentiating BP from another major entity that causes the appearance of consolidation in imaging tests, atelectasis. It will be conducted at Sant Joan de Déu Hospital in Spain from June 2025 to June 2027. Firstly, imaging biomarkers will be measured in well-aerated lung tissue without consolidation to establish their values in healthy lung tissue, according to a predefined imaging acquisition protocol. Subsequently, the imaging biomarkers will be assessed in patients with confirmed lung consolidation by LUS (Group 1: BP; Group 2: atelectasis). **Results**: The study aims to determine whether there are statistically significant differences in the biomarker values in relation to the normal values and between the different etiological groups. **Conclusions**: The demonstration of the reliable diagnostic accuracy of these biomarkers could significantly reduce the need for invasive techniques and improve the therapeutic management of many patients with BP and other pulmonary conditions presenting with consolidation in imaging tests.

## 1. Introduction

Acute lower respiratory tract infections are highly prevalent in the paediatric population and represent one of the leading causes of admission to paediatric intensive care units (PICUs) [1]. Specifically, community-acquired pneumonia (CAP) remains one of the primary causes of death among paediatric patients worldwide [2]. The typical symptoms of pneumonia are fever, cough, tachypnoea, and respiratory distress. However, younger children may present with non-specific symptoms such as irritability or refusal to feed [3]. CAP is primarily caused by bacterial or viral infections. Although the diagnosis of CAP generally relies on a combination of clinical signs, blood tests, imaging techniques, and microbiological parameters, the British Thoracic Society (BTS) recommends basing the diagnosis on clinical findings alone, advising against routine use of chest X-rays (CXRs) to reduce radiation exposure. Furthermore, the use of acute phase reactants to differentiate between viral and bacterial infections is discouraged [4]. In the PICU setting, nosocomial pneumonia (NP) due to bacterial infection is particularly common. This category includes both hospital-acquired pneumonia and ventilator-associated pneumonia (VAP) and constitutes one of the main healthcare-associated infections in hospitals [5]. NP should be suspected in any patient who develops a lung infection after 48 h of hospitalization or within the first 7 days following hospital discharge. Focusing on VAP, it develops after 48 h of mechanical ventilation and is identified by the presence of a new infiltrate on CXR, along with positive microbiological findings from the lower respiratory tract and/or two or more positive clinical signs of pneumonia. VAP is a highly prevalent condition, and while there is no single Gold Standard diagnostic tool, the Centers for Disease Control and Prevention (CDC) criteria are the most widely accepted and utilized [6].

Regarding the use of analytical parameters, the British Thoracic Society (BTS) discourages the routine use of acute phase reactants, such as C-reactive protein (CRP) or procalcitonin (PCT), for differentiating between bacterial and viral aetiologies [4]. The Infectious Diseases Society of America (IDSA) recommends using these markers alongside clinical parameters only in patients with severe infections requiring hospitalization or those who develop pneumonia-related complications [7]. Microbiological data can assist in confirming a potential infectious aetiology; however, it is often unavailable during the early stages of diagnosis. Additionally, sputum cultures, blood cultures, and bronchoalveolar lavage exhibit relatively low sensitivity and do not always identify the causative agent [8]. In contrast, recent advancements in PCR technology have enabled the detection of multiple specific respiratory pathogens, including both viruses and bacteria, achieving high diagnostic accuracy [9,10]. Regarding imaging tests, IDSA guidelines recommend performing a CXR with both posteroanterior and lateral projections in all children hospitalized with pneumonia who present hypoxemia or respiratory distress, as well as in cases where initial antibiotic therapy fails [7]. The characteristic radiographic finding of BP is the presence of a consolidation (lobar pattern). However, this is not a pathognomonic feature of BP and may be associated with other non-infectious conditions, the most common of which is atelectasis. Atelectasis is defined as the partial or total collapse of a lung segment, which can result from internal airway obstruction (e.g., due to mucus), extrinsic compression (e.g., from a pleural effusion or pneumothorax), or a loss of surfactant (particularly relevant in premature infants). This condition can impair gas exchange and lead to symptoms similar to those caused by pneumonia, including respiratory distress, cough, and/or chest pain. Less commonly, lung consolidation may be due to pulmonary contusions or pulmonary thromboembolism, although the patient’s clinical context is usually characteristic and facilitates its identification [11].

**Figure 1:**
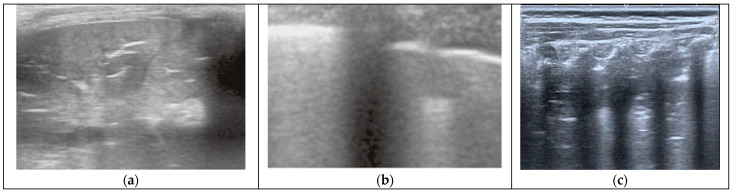
(**a**) BP as seen by LUS, showing a large consolidation with branched air bronchograms inside. (**b**) VP seen by LUS, showing coalescent B-lines diffusely along with small, multiple subpleural consolidations. (**c**) Atelectasis seen by LUS, showing a large area of consolidation with parallel air bronchograms (non-dynamic).

In recent years, lung ultrasound (LUS) has been increasingly used to enhance the pneumonia diagnosis. LUS is a reliable, rapid, and non-invasive technique that can be performed at the patient’s bedside. Moreover, LUS has reduced radiological exposure associated with the routine use of CXR and has decreased antibiotic exposure [12,13]. In most cases, LUS may allow the distinction between BP and VP. The typical LUS findings of BP are pleural line disruption, a hypoechoic consolidation (>1 cm) with arboriform and dynamic air bronchogram inside, and perilesional B-line enhancement, often associated with pleural effusion (Figure 1a). In contrast, VP is usually seen on LUS as small subpleural consolidations (<1 cm) and accumulation of perilesional B-lines, without air bronchogram (Figure 1b). Atelectasis typically appears on LUS as an area of consolidated tissue (>1 cm) with a static and parallel bronchogram and possible loss of movement of the adjacent pleura (Figure 1c) [14,15,16]. Several publications have demonstrated the high efficacy of LUS in diagnosing these conditions compared to CXR. Yan et al. found that the pooled sensitivity, specificity, and diagnostic odds ratio (DOR) for paediatric pneumonia diagnosed by LUS were 0.95 (95% CI: 0.94 to 0.96), 0.90 (95% CI: 0.87 to 0.92), and 137.49 (95% CI: 60.21 to 313.98), respectively. For CXR, the pooled sensitivity, specificity, and DOR were 0.91 (95% CI: 0.90 to 0.93), 1.00 (95% CI: 0.99 to 1.00), and 369.66 (95% CI: 137.14 to 996.47), respectively. Similarly, several studies have assessed the utility and effectiveness of LUS in diagnosing atelectasis compared to CXR [17]. Martinez-Molina et al. compared both imaging techniques in 100 adult patients and found that LUS demonstrated a sensitivity of 85%, specificity of 100%, positive predictive value of 100%, and negative predictive value of 55% for detecting atelectasis [18].

Given all this, in daily clinical practice, particularly in hospital wards or the PICU, the main difficulty for clinicians lies in the differentiation between BP and atelectasis, due to the similarity of their LUS findings in patients with possible similar clinical features. This is especially relevant in situations where BP has minimal impact and no fever or signs of infection are present during the patient’s physical examination. In contrast, VP is usually well differentiated through LUS and the clinical context, as are other conditions such as contusion or pulmonary thromboembolism, which can also cause lung consolidations but are typically framed within a very specific and characteristic clinical context.

Given the difficulty of LUS in some cases in differentiating between BP and atelectasis, a precise differential diagnosis of these conditions often requires invasive procedures, such as blood draws, which can be particularly challenging in paediatrics. Previous studies, such as the PROLUSP trial, have demonstrated that combining LUS with PCT enhances diagnostic accuracy for BP, achieving 90% sensitivity and 85% specificity [19,20]. Our research group suggests evaluating the usefulness of new, quantitative, non-invasive, and reproducible imaging biomarkers for diagnosing BP and atelectasis, such as lung elastance measured by Shear Wave Elastography (SWE) and the lung microvascular ratio assessed by Superb Microvascular Imaging (SMI). SWE assesses tissue elastance by measuring the propagation speed of shear waves, which originate perpendicularly from the initial acoustic radiation emitted by the transducer as it scans multiple focal areas of the tissue [21,22,23]. SWE is widely used for the liver, breast, prostate, and thyroid, but its application to lung tissue remains limited, despite several research groups suggesting its potential usefulness in this organ [24,25,26,27]. Similarly, the role of microvascularization within a pulmonary consolidation has not been studied to date, so its relevance in the differential diagnosis of various lung consolidations remains unknown. In recent years, technological tools have been developed and improved to allow the visualization of blood flow in microvessels. Specifically, SMI technology can suppress interference and thereby extract the blood flow signal from both large and small vessels, ultimately representing this information as a color-overlay image and translating it into a microvascularization ratio. Its use has been studied mainly in the breast and thyroid, so knowledge of its usefulness in the lung is very scarce [28,29].

Table 1 provides a comparison of the characteristics of the different imaging techniques mentioned, based on their applicability to lung tissue.

Considering that in BP, inflammation and alveolar filling with fluid and immune cells occur, leading to the hepatization of pulmonary tissue, while in atelectasis, there is alveolar collapse with a reduction in lung volume and tissue compression, our research group hypothesizes that BP will exhibit decreased pulmonary elasticity but a higher microvascularization ratio compared to atelectasis. Therefore, both SWE and SMI may play a crucial role in assessing these pathophysiological characteristics, thus contributing to the accurate characterization and diagnosis of these conditions. This hypothesis is supported by several studies conducted in adults. Lim et al. evaluated the elasticity of atelectasis and BP, among other lesions, and reported an elasticity of 2.51 ± 1.14 kPa in atelectasis and 19.98 ± 15.59 kPa in pneumonic consolidations. To date, no studies have been published regarding the microvascularization ratio in these lesions [23].

Up until now, the normal values of these imaging biomarkers and the role of these diagnostic tests have yet to be evaluated in the paediatric population. The ELASMIC study aims to analyze the ability of quantitative lung imaging biomarkers (elastance measured with SWE and microvascularization ratio measured with SMI) to discriminate between BP from other causes of lung consolidation such as atelectasis, reducing the need for invasive and radiation-based diagnostics in the paediatric population. As discussed previously, distinct values of elastance and microvascularization ratio are expected given the pathophysiological differences of each entity. The identification of statistically significant differences in imaging biomarkers between BP and atelectasis could significantly enhance the diagnostic approach to these disorders, offering a less invasive method and enabling a more precise quantitative characterization of the underlying pathological processes.

## 2. Materials and Methods

### 2.1. Study Design

**Figure 2:**
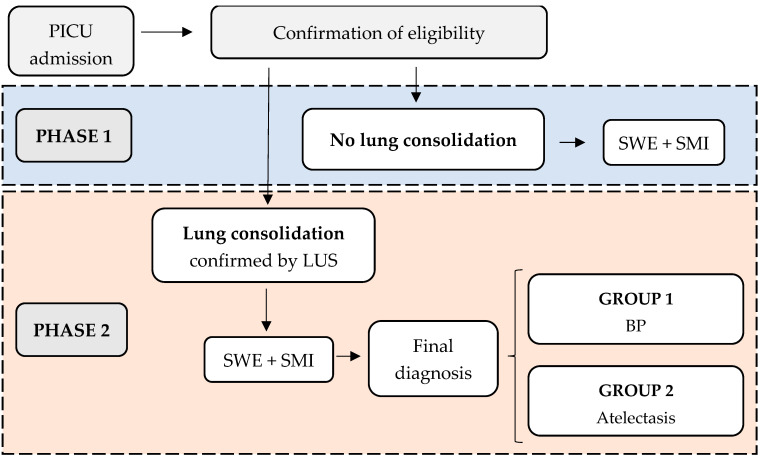
ELASMIC study phases. PICU: Paediatric Intensive Care Unit; LUS: Lung Ultrasound; SWE: Shear Wave Elastography; SMI: Superb Microvascular Imaging; BP: Bacterial Pneumonia.

This is a prospective cohort study aimed at validating the effectiveness of two novel non-invasive imaging biomarkers. It will be conducted at Sant Joan de Déu Hospital, a tertiary children’s hospital in Barcelona (Spain). The recruitment and follow-up period is planned from June 2025 to June 2027. As previously mentioned, the imaging acquisition protocol will guide the various phases of the study (Figure 2):

Phase 1: Measurement of elastance and microvascularization ratio in well-aerated (non-consolidated) lung tissue. Patients without pulmonary consolidation will be included in this phase.

Phase 2: Measurement of elastance and microvascularization ratio in consolidated lung tissue. Patients with confirmed pulmonary consolidation via LUS (even if the initial detection was through other imaging techniques) will be recruited. Patients will then be classified into two groups based on the final diagnosis:

- Group 1: BP
- Group 2: Atelectasis

The final BP diagnosis will be made by a clinical expert using the BTS definition for CAP and the CDC criteria for VAP. This includes compatible clinical symptoms (such as fever, cough, tachypnea, and shortness of breath), abnormal auscultation patterns, LUS findings (according to Section 2.4.1), and blood test abnormalities suggestive of bacterial infection (leukocytosis, neutrophilia, CRP > 50 mg/L, and/or PCT > 1 ng/mL). Atelectasis will be diagnosed in patients with respiratory distress, without blood test abnormalities suggestive of bacterial infection (CRP < 50 mg/L and PCT < 1 ng/mL), and compatible LUS findings (according to Section 2.4.1), which resolve after increasing ventilatory support and performing respiratory physiotherapy.

For the differential diagnosis of both entities, respiratory sample cultures will not be considered, as false negatives may occur (in the context of BP) or colonizing organisms may be detected (even in cases of atelectasis).

### 2.2. Outcomes

1. Determine normal values for the imaging biomarkers (elastance and microvascularization) in healthy lung parenchyma and within different healthy areas.
2. Determine the values for the imaging biomarkers (elastance and microvascularization) in lung consolidation.
3. Evaluate differences between the imaging biomarker values on healthy lung vs. consolidated lung.
4. Evaluate differences between the imaging biomarker values of group 1 (BP) vs. group 2 (atelectasis). This would allow the establishment of a cut-off point for the etiological differentiation of lung consolidations.

### 2.3. Inclusion and Exclusion Criteria

Inclusion: children under 18 years of age admitted to the hospital without pulmonary pathology will be recruited for developing phase 1 of the study. Subsequently, children under 18 years of age admitted to the PICU with lung consolidation present on admission or developed during their stay will be recruited for developing phase 2 of the study. Signed acceptance of informed consent must be obtained from the children’s legal guardians.

Exclusion: children with a history of pulmonary (asthma, bronchopulmonary dysplasia, pulmonary fibrosis, etc.), oncological, or cardiac disorders, those in an immunosuppressed state, or those in end-of-life care will be excluded. This aims to reduce the possibility of introducing artifacts in the results due to the inclusion of patients with a potential baseline alteration of the biomarkers being studied. Additionally, patients participating in other clinical trials, as well as those who refuse to sign the informed consent form or withdraw consent later, will also be excluded from the study.

### 2.4. Imaging Acquisition Protocol

The LUS image acquisition protocol was developed to characterize and identify lung consolidation, as well as to integrate SWE and SMI lung measurements. This aims to standardize imaging procedures and reduce operator variability. The study will use a high-resolution linear ultrasound device (Aplio A500 Canon™; Tokyo, Japan) with an acoustic frequency of 7–14 MHz. If the linear probe does not allow visualization of up to 3 cm of pulmonary tissue depth (likely due to significant adipose tissue in the exploration area), a convex probe with a 5 MHz frequency will be used. This situation is expected to be exceptional, as we are evaluating paediatric patients, in whom pulmonary tissue is typically well visualized with the linear probe. Therefore, the collected data will be evaluated in a combined manner without distinguishing between the probes used. The patient will be evaluated in a supine position. In case of impossibility, the assessment will be conducted in a seated position.

#### 2.4.1. Lung Ultrasound Imaging Acquisition

LUS evaluation will be performed at the bedside, adhering to standard clinical practice. Lung consolidation will be identified by liver-like images or parenchymal images within the lung tissue with dynamic or non-dynamic bronchogram, anfractuous edges, and “shred” signs.

The LUS findings of BP include:

- Disruption of the pleural line
- Hypoechoic consolidation with dynamic and arborizing air bronchogram
- Perilesional B-line enhancements.

The LUS findings of atelectasis include:

- Hypoechoic consolidation with non-dynamic and parallel air bronchogram
- Possible loss of pleural movement adjacent to the consolidation.

#### 2.4.2. Lung Shear Wave Elastography Acquisition

**Figure 3:**
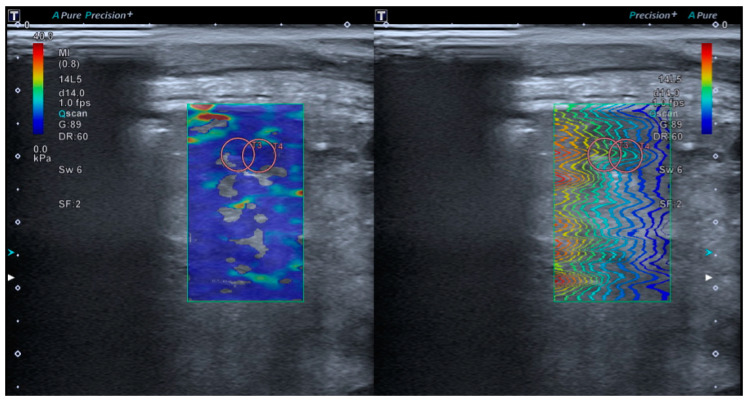
The TwinView feature of the Aplio A500 device (Canon™) allows for the visualization of the elastance map (**left**) and the propagation map (**right**).

Tissue elastance will be measured using lung SWE through an intercostal window, minimizing the manual pressure applied by the transducer. The SWE ‘Multi’ mode (Real-Time Shear Wave acquisition) will be activated, triggering a dual screen (TwinView), where the elastance map will be displayed on the left and the propagation map on the right (Figure 3). A colored box will be placed 1 cm from the capsule on the elastance map (with an elastance scale set at 40 kPa). Subsequently, a circular Region of Interest (ROI) of 10 mm (size 5 on our device) will be selected, with a measurement depth limited to a maximum of 5 cm. Measurements will preferably be taken in areas with homogeneous color on the elastance map and with shear wave propagation as parallel as possible. Of all the measurements taken, the two with the lowest standard deviation and an interquartile range (IQR) < 0.3 will be selected, and their mean value will be used for inclusion in the final statistical analysis.

#### 2.4.3. Superb Microvascular Imaging Acquisition

**Figure 4:**
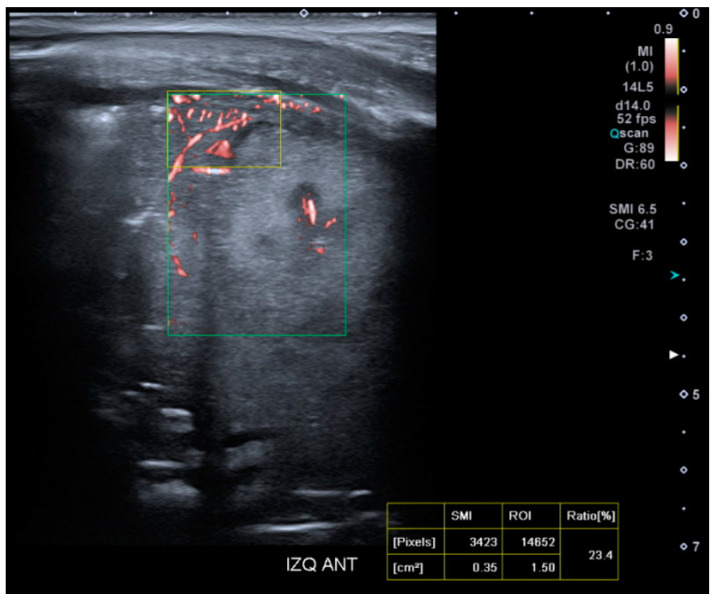
Presentation interface of the SMI mode on the Aplio A500 device (Canon™).

For the study of tissue microvascularization, the transducer will be kept perpendicular to the skin, using the same intercostal window without modifying the patient’s position. The microvascularization ratio measurement will be performed using SMI within the colored box previously defined for lung SWE measurement (Figure 4). The SMI Doppler mode will be activated, along with the ‘Vascular Index’ tool, selecting a velocity scale of 0.9 m/s. A rectangular ROI (15 × 10 mm) will be selected at a maximum depth of 5 cm. Microvascularization results will be expressed as a percentage.

### 2.5. Variables

Demographic, clinical, analytical, microbiological, ultrasound, SWE, and SMI variables will be collected (Table 2).

### 2.6. Ethical Considerations and Data Collection

The study will be conducted in accordance with the ICH E11 Guideline and the Declaration of Helsinki (version Fortaleza, Brasil 2013). The study protocol has been approved by the Ethics Committee of Sant Joan de Déu Hospital. Patients will be enrolled by the study investigators after verifying that all inclusion criteria are met. The confidentiality of patient identities will be maintained throughout the study, and all data will be handled in compliance with Organic Law 3/2018 on the Protection of Personal Data. In accordance with this law, only data necessary to achieve the study’s objectives will be collected. Given that the study population will consist of critically ill paediatric patients, informed consent will be obtained from the legal representatives of these patients in all cases. Complete identifying information and written consent will be securely stored in the researcher’s files. A REDCAP database has been developed and securely hosted in a restricted-access cloud environment. This database includes automatic inconsistency detection filters to ensure data accuracy and provides full traceability of all recorded data. Access to the database will be limited to physicians and clinical researchers directly involved in the study, the Ethics Committee, and relevant health authorities, ensuring strict compliance with regulatory and ethical standards.

### 2.7. Statistical Analysis and Sample Size

To determine the normal reference values of elastance and microvascularization ratio in the healthy lung, measures of central tendency (mean and median) and dispersion (standard deviation, interquartile range) will be calculated for both elastance and microvascularization variables. The distribution of the variables will be assessed using the Shapiro–Wilk test to determine if the data follows a normal distribution. If the variables follow a normal distribution, the standard approach will be applied using the Faulkenberry-Weeks approach implemented in the tolerance R package. If the variables deviate from normality, non-parametric bootstrap methods will be used to estimate the tolerance intervals. Variability of the biomarkers across different patient subgroups (e.g., by sex or age) will be explored using analysis of variance (ANOVA), provided the assumptions of ANOVA are met. If assumptions are not met, permutation tests will be applied. A *p*-value of less than 0.05 will be considered statistically significant for all statistical tests. A sample of 60 subjects is considered sufficient to obtain 90% tolerance intervals at 95% confidence with a 5% error in tolerance and a 2.5% error in confidence [30].

Subsequently, to establish differences in the values of the imaging biomarkers among the different groups (BP, atelectasis, and normal baseline), analysis of variance (ANOVA) will be performed. In the event of finding significant differences, post-hoc analysis will be conducted using Tukey’s method. If the conditions for the applicability of ANOVA are not met, a permutation test will be applied. The sample size for the ANOVA test is calculated using the method proposed by Cohen. According to this method, the test effect size is the ratio of the sum of squares between groups to the total sum of squares. Cohen suggested a value of 0.25 as a medium effect size. The sample size is calculated using the pwr R package, which gives a sample size of 53 subjects per group required to detect a medium effect size with 80% power and three groups [31].

## 3. Limitations

One of the main limitations of this project is that it is a single-centre study. However, it is a tertiary paediatric hospital that handles a high volume of patients, making the sample potentially large and representative at the population level. A potential selection bias in Phase 1 may arise from evaluating imaging biomarkers in patients who present to the hospital for reasons unrelated to pulmonary consolidation. This could limit the generalization of normal value results to the general population. Another limitation of the study is the potential for artifacts in biomarker measurements due to the presence of air in non-consolidated pulmonary tissue. If this issue occurs, it cannot be addressed by adjustments to the biomarker acquisition technique and would affect Phase 1 of the study. In this phase, it may be concluded that SWE and SMI are not reliable pulmonary diagnostic tools in the absence of consolidation. This could serve as a key preliminary finding of the study. In contrast, in Phase 2, where consolidated (non-aerated) pulmonary tissue is being assessed, the presence of air is unlikely to cause measurement artifacts. Another relevant aspect is the potential inter-observer variability, although this is not included among the objectives of the ELASMIC study, and thus, imaging biomarkers will be acquired by a single examiner. However, given that the examiner is a highly qualified professional trained in LUS image acquisition, the study assumes minimal inter-observer variability (the differences in measurements taken by another observer are negligible and clinically insignificant) and minimal intra-observer variability (the differences in measurements taken by the same examiner are negligible and clinically insignificant). Another limitation, which also represents a strength due to its innovative potential, is the use of diagnostic techniques that are not conventionally employed for evaluating lung tissue. In this regard, significant differences in the manual pressure exerted by the operator with the transducer could modify the measured results. Therefore, it will be essential to apply minimal pressure and verify the stability of the color map prior to the acquisition of the biomarkers.

## 4. Discussion

Pulmonary pathology is highly prevalent in paediatrics, especially in the PICU setting. The differential diagnosis between various pulmonary conditions, such as BP and atelectasis, is crucial to provide the best therapy approach for each patient, limiting antibiotic use to those who truly need it, thus preventing overexposure to antibiotics and the rise of antimicrobial resistance. Currently, the differential diagnosis requires invasive procedures such as analytical extractions and imaging tests like CXR, which involve a high radiation burden. In this regard, bedside LUS has been widely introduced into daily clinical practice in recent years, and our research group has published several studies on its application in paediatrics and its impact on the etiological approach and therapeutic decision-making. In the study by Haakma et al., conducted in an adult ICU, it was concluded that the presence of dynamic air bronchogram in LUS exhibited a sensitivity of 45% (95% CI, 31–60%) and a specificity of 99% (95% CI, 92–100%) for BP diagnosis, while the presence of Colour Doppler imaging demonstrated a sensitivity of 90% (95% CI, 79–97%) and a specificity of 68% (95% CI, 56–79%) [11]. Therefore, there remains an opportunity to improve the diagnosis of BP and other causes of pulmonary consolidation through quantifiable and non-invasive biomarkers.

In this context, through this study protocol, we propose the investigation of new quantifiable and reproducible imaging biomarkers that provide individualized results and can be obtained non-invasively, aiming to improve the quality of care with a gender perspective and focused on the well-being of the patient and their family. Specifically, we suggest studying lung elastance measured by SWE and microvascularization ratio measured by SMI. The incorporation of these new techniques would represent a significant change in the diagnostic approach for patients with BP and atelectasis [32,33,34].

ELASMIC aims to update and enhance current protocols for diagnosing BP and other pulmonary consolidations. Specifically, it seeks to reduce the reliance on CXR while maintaining the current role of LUS in detecting various pulmonary consolidations. Furthermore, SWE and SMI could play a crucial complementary role to LUS in characterizing these pulmonary consolidations and providing etiological guidance. To achieve this, it is essential to determine the normal values for lung elastance and microvascular patterns in healthy lung tissue. These baseline values will enable the assessment of non-invasive biomarkers in patients with lung consolidation, ultimately facilitating differentiation between various potential causes. Standardized image acquisition and biomarker measurement will help reduce operator-dependent variability. Despite the limitations mentioned, achieving the study’s objectives could significantly improve clinical management through a quantifiable and non-invasive approach. Moreover, it may optimize therapeutic strategies by reducing the use of empirical antibiotics in patients without bacterial infections, which could have a substantial positive impact on global health.

## Figures and Tables

**Table 1:** Comparison of the characteristics of CXR, LUS, SWE, and SMI for the evaluation of pulmonary pathology.

	CXR	LUS	SWE	SMI
**Irradiation**	Yes	No	No	No
**Bedside**	No	Yes	Yes	Yes
**Reproducibility**	Not operator-dependent	Operator-dependent	Operator-dependent	Operator-dependent
**Quantitative data**	No	No	Yes	Yes
**Type of information it provides**	Anatomy overview and possible pulmonary pathologies	Pleural effusions, pneumothorax, or superficial lung consolidations	Elasticity of the lung tissue	Lung microvascularization, (perfusion and small blood vessels)

CXR: Chest X-Ray; LUS: Lung Ultrasound; SWE: Shear Wave Elastography; SMI: Superb Microvascular Imaging.

**Table 2:** Set of variables to be collected during the study.

	Variables
**Anthropometric and Demographic**	- Age - Sex - Weight - Ethnicity
**Clinical and analytical**	- Reason for admission to PICU (if admitted) - Heart rate, respiratory rate, blood pressure and oxygen transcutaneous saturation and pulmonary auscultation findings. - Presence of respiratory failure criteria. - PRISM-III. - Analytical parameters (leukocyte count, CRP, PCT). - Aetiological study in biological samples collected. - Antibiotic treatment received. - Final diagnosis
**Respiratory**	- Need and type of mechanical ventilation (invasive/non-invasive) - Duration of mechanical ventilation - Respiratory pattern (obstructive/restrictive) - Lung compliance and resistance values (if invasive ventilation) - Radiological findings (if CXR performed)
**Ultrasound**	- Presence of A and/or B lines. - Presence of lung sliding. - Presence and description of pulmonary consolidations. - Presence of air bronchogram (position/type). - Assessment of the pleural space (free/fluid occupancy). - Presence of pneumothorax.
**SWE**	- Lung elastance values (KPa and m/s) in liver, healthy lung and in pulmonary condensation - Quantification of microvascularization through SMI (ratio) in healthy lung and in lung consolidation.

PICU: Paediatric Intensive Care Unit; PRISM-III: Paediatric Risk of Mortality; CRP: C-reactive protein; PCT: procalcitonin; CXR: Chest X-ray; SWE: Shear Wave Elastography; SMI: Superb Microvascular Imaging.

## Data Availability

The original contributions presented in this study are included in the article. Further inquiries can be directed to the corresponding author.

## References

[1] Koh J.W.J.C., Wong J.J.-M., Sultana R., Wong P.P.C., Mok Y.H., Lee J.H. (2017). Risk Factors for Mortality in Children with Pneumonia Admitted to the Pediatric Intensive Care Unit: Risk Factors for Mortality in Pediatric Pneumonia. Pediatr. Pulmonol..

[2] Unicef (2015). Committing to Child Survival: A Promise Renewed: Progress Report 2015.

[3] Shah S.N., Bachur R.G., Simel D.L., Neuman M.I. (2017). Does This Child Have Pneumonia?: The Rational Clinical Examination Systematic Review. JAMA.

[4] Harris M., Clark J., Coote N., Fletcher P., Harnden A., McKean M., Thomson A., On behalf of the British Thoracic Society Standards of Care Committee (2011). British Thoracic Society Guidelines for the Management of Community Acquired Pneumonia in Children: Update 2011. Thorax.

[5] Iosifidis E., Pitsava G., Roilides E. (2018). Ventilator-Associated Pneumonia in Neonates and Children: A Systematic Analysis of Diagnostic Methods and Prevention. Future Microbiol..

[6] National Healthcare Safety Network—CDC (2025). Pneumonia (Ventilator-Associated [VAP] and Non-Ventilator- Associated Pneumonia [PNEU]) Event. https://www.cdc.gov/nhsn/pdfs/pscmanual/6pscvapcurrent.pdf.

[7] Bradley J.S., Byington C.L., Shah S.S., Alverson B., Carter E.R., Harrison C., Kaplan S.L., Mace S.E., McCracken G.H., Moore M.R. (2011). The Management of Community-Acquired Pneumonia in Infants and Children Older Than 3 Months of Age: Clinical Practice Guidelines by the Pediatric Infectious Diseases Society and the Infectious Diseases Society of America. Clin. Infect. Dis..

[8] Bartlett J.G., Breiman R.F., Mandell L.A., File T.M. (1998). Community-Acquired Pneumonia in Adults: Guidelines for Management. Clin. Infect. Dis..

[9] Serigstad S., Knoop S.T., Markussen D.L., Ulvestad E., Bjørneklett R.O., Ebbesen M.H., Kommedal Ø., Grewal H.M.S. (2023). Diagnostic Utility of Oropharyngeal Swabs as an Alternative to Lower Respiratory Tract Samples for PCR-Based Syndromic Testing in Patients with Community-Acquired Pneumonia. J. Clin. Microbiol..

[10] Demars Y., Brahier T., Rotzinger D.C., Brouillet R., Jaton K., Opota O., Boillat-Blanco N. (2022). Utility of Polymerase Chain Reaction in Nasopharyngeal Swabs for Identifying Respiratory Bacteria Causing Community-Acquired Pneumonia. Microbiol. Spectr..

[11] Haaksma M.E., Smit J.M., Heldeweg M.L., Nooitgedacht J.S., De Grooth H.J., Jonkman A.H., Girbes A.R., Heunks L., Tuinman P.R. (2022). Extended Lung Ultrasound to Differentiate Between Pneumonia and Atelectasis in Critically Ill Patients: A Diagnostic Accuracy Study. Crit. Care Med..

[12] Jones B.P., Tay E.T., Elikashvili I., Sanders J.E., Paul A.Z., Nelson B.P., Spina L.A., Tsung J.W. (2016). Feasibility and Safety of Substituting Lung Ultrasonography for Chest Radiography When Diagnosing Pneumonia in Children. Chest.

[13] Guitart C., Bobillo-Perez S., Rodríguez-Fanjul J., Carrasco J.L., Brotons P., López-Ramos M.G., Cambra F.J., Balaguer M., Jordan I. (2024). Lung Ultrasound and Procalcitonin, Improving Antibiotic Management and Avoiding Radiation Exposure in Pediatric Critical Patients with Bacterial Pneumonia: A Randomized Clinical Trial. Eur. J. Med. Res..

[14] Buonsenso D., Musolino A., Ferro V., De Rose C., Morello R., Ventola C., Liotti F.M., De Sanctis R., Chiaretti A., Biasucci D.G. (2022). Role of Lung Ultrasound for the Etiological Diagnosis of Acute Lower Respiratory Tract Infection (ALRTI) in Children: A Prospective Study. J. Ultrasound.

[15] Varshney T., Mok E., Shapiro A.J., Li P., Dubrovsky A.S. (2016). Point-of-Care Lung Ultrasound in Young Children with Respiratory Tract Infections and Wheeze. Emerg. Med. J..

[16] Shah V.P., Tunik M.G., Tsung J.W. (2013). Prospective Evaluation of Point-of-Care Ultrasonography for the Diagnosis of Pneumonia in Children and Young Adults. JAMA Pediatr..

[17] Yan J.-H., Yu N., Wang Y.-H., Gao Y.-B., Pan L. (2020). Lung Ultrasound vs. Chest Radiography in the Diagnosis of Children Pneumonia: Systematic Evidence. Medicine.

[18] Martínez-Molina J.-A., Martínez-González M.A., Vives Santacana M., González Delgado A.D., Reviejo Jaka K., Monedero P. (2024). Diagnostic comparison of bedside lung ultrasound and chest radiography in the intensive care unit. An. Sist. Sanit. Navar..

[19] Rodríguez-Fanjul J., Guitart C., Bobillo-Perez S., Balaguer M., Jordan I. (2020). Procalcitonin and Lung Ultrasound Algorithm to Diagnose Severe Pneumonia in Critical Paediatric Patients (PROLUSP Study). A Randomised Clinical Trial. Respir. Res..

[20] Alejandre C., Balaguer M., Guitart C., Torrús I., Felipe A., Launes C., Cambra F.J., Jordan I. (2020). Procalcitonin-guided Protocol Decreased the Antibiotic Use in Paediatric Patients with Severe Bronchiolitis. Acta Paediatr..

[21] Huerta-Calpe S., Salas B., Inarejos Clemente E.J., Guitart C., Balaguer M., Jordan I. (2023). Sono-Elastography: An Ultrasound Quantitative Non-Invasive Measurement to Guide Bacterial Pneumonia Diagnosis in Children. Children.

[22] Sigrist R.M.S., Liau J., Kaffas A.E., Chammas M.C., Willmann J.K. (2017). Ultrasound Elastography: Review of Techniques and Clinical Applications. Theranostics.

[23] Lim C.-K., Chung C.-L., Lin Y.-T., Chang C.-H., Lai Y.-C., Wang H.-C., Yu C.-J. (2017). Transthoracic Ultrasound Elastography in Pulmonary Lesions and Diseases. Ultrasound Med. Biol..

[24] Ricci P., Maggini E., Mancuso E., Lodise P., Cantisani V., Catalano C. (2014). Clinical Application of Breast Elastography: State of the Art. Eur. J. Radiol..

[25] Correas J.-M., Tissier A.-M., Khairoune A., Vassiliu V., Méjean A., Hélénon O., Memo R., Barr R.G. (2015). Prostate Cancer: Diagnostic Performance of Real-Time Shear-Wave Elastography. Radiology.

[26] Asteria C., Giovanardi A., Pizzocaro A., Cozzaglio L., Morabito A., Somalvico F., Zoppo A. (2008). US-Elastography in the Differential Diagnosis of Benign and Malignant Thyroid Nodules. Thyroid.

[27] Huerta-Calpe S., Guitart C., Salas B., Cambra F.J., Jordan I., Balaguer M. (2024). Use of Non-Invasive Elastographic and Microvascularization Biomarkers in the Diagnosis and Follow-Up of Children with Severe Bacterial Pneumonia. SciBase Clin. Med. Case Rep..

[28] Feng J., Lu J., Jin C., Chen Y., Chen S., Guo G., Gong X. (2022). Diagnostic Value of Superb Microvascular Imaging in Differentiating Benign and Malignant Breast Tumors: A Systematic Review and Meta-Analysis. Diagnostics.

[29] Jin H., Wang C., Jin X. (2022). Superb Microvascular Imaging for Distinguishing Thyroid Nodules: A Meta-Analysis (PRISMA). Medicine.

[30] Faulkenberry G.D., Weeks D.L. (1968). Sample Size Determination for Tolerance Limits. Technometrics.

[31] Cohen J. (2013). Statistical Power Analysis for the Behavioral Sciences.

[32] Zhang X., Qiang B., Hubmayr R.D., Urban M.W., Kinnick R., Greenleaf J.F. (2011). Noninvasive Ultrasound Image Guided Surface Wave Method for Measuring the Wave Speed and Estimating the Elasticity of Lungs: A Feasibility Study. Ultrasonics.

[33] Zhang X., Osborn T., Zhou B., Meixner D., Kinnick R.R., Bartholmai B., Greenleaf J.F., Kalra S. (2017). Lung Ultrasound Surface Wave Elastography: A Pilot Clinical Study. IEEE Trans. Ultrason. Ferroelect. Freq. Contr..

[34] Jiang Z., Huang Y., Shen H., Liu X. (2019). Clinical Applications of Superb Microvascular Imaging in the Liver, Breast, Thyroid, Skeletal Muscle, and Carotid Plaques. J. Ultrasound Med..

